# Supplementary material for: Psychometric properties of the Malay version of the difficulties in Emotion Regulation Scale-18 in Malaysian adolescents
Source: PLoS One. 2023 Aug 28;18(8):e0289551. doi: 10.1371/journal.pone.0289551 (PMC10461853; doi:10.1371/journal.pone.0289551)
Supplement: Attachment — Submitted filename: Response to Reviewers.pdf [file pone.0289551.s002.pdf]

Dear Reviewers,

Thank you for taking the time to review our manuscript. We appreciate your valuable comments and suggestion, which have helped us to improve the quality of our manuscript. Your feedback has been very insightful and we are grateful for your time and effort in reviewing our work.

We have addressed each of your suggestions and made amendments accordingly. The amendments are presented on the following pages. Once again, thank you for your valuable input. We hope that the revised manuscript meets your expectations and look forward to your feedback.

Sincerely,

Authors

Reviewer 1

	Comment	Amendment
1.	There are a few grammatical errors, incorrect spacing, and a full stop throughout the paper.	We have sent the manuscript for proofreading and editing
2.	There was inconsistency regarding the study sample size. For instance, 701, 700, and 689	The size sample was different at the data collection (701 participants) and at the final analysis (689 participants) because of missing data<br><br>We added a clarification at line 197, 217 to 219, and 387 to 390.
3.	The paragraph on obtaining ethical approval should be under the heading "Ethical approval."	Changes have been made at line, see line 297 to 300<br><br>From line 308 to 317 is the paragraph on obtaining ethical approval
4.	The authors stated that "A random stratified sampling was used to select the schools from each region." The authors should provide some information about this process.	We added a clarification from line 203 to 212.

Reviewer 2

	Comment	Amendment
**Abstract**	This section in all subsection should be revised and completed according to the following suggestions, for example methods subsection is not complete and you mentioned partly some aspects of psychometric analysis done on needed to be done.	We have added analysis procedure<br><br>See line 33 to 37
	I am not agree with predictive validity, it is criterion validity (divergent/convergent validity)	The word predictive have been replaced with criterion (concurrent and predictive)<br><br>See line 50
	How about the test retest reliability results?	This study was conducted during the Covid-19 pandemic, the adolescents emotional state may not be stable would be affected by the unprecedented event over a period of time. We did not perform the test-retest study (that is mainly to look into the replicability of the responses) as we felt it is not applicable for the current study. Hence the result is not available.<br><br>In addition, as the data collection was conducted during restricted Movement Control Order of the Covid-19 pandemic, it was difficult to ensure to catch the same students for the re-test session as some students may be absent from the (online or physical) classes because of health reasons, school truancy or internet issues. Their academic activities were very much affected and it would be difficult for us to arrange meetings of more than once as the priority was more on more on completing their syllabus.

	The presented data about criterion validity is not correct,	We have made the correction. We subdivide the criterion validity into concurrent validity and predictive validity<br><br>See line 44 to 48
	why r-squared not r and	The reason is that previous studies had examined the predictive validity of the DERS-18 using regression analysis. Thus, we aimed to replicate the findings
	how about p-value for r.	We added the results of r and p-value<br><br>See line 44 to 46
	Conclusion does not present any thing about construct validity.	Have added in the conclusion<br><br>See line 50
	not suitable keyword :Malay validation, validity or psychometric analysis, you can include Malesia as a keyword separately, use construct validity instead of confirmatory factor analysis	Changes have been made<br><br>See line 53 to 54
**Intro**	Introduction is very long, should be effectively shorten with more focuses on conducted studies worldwide about validity and reliability of DERS-18	We have shortened the introduction, while retaining important information such as the dimensionality and factor structure of the DERS-18. We believe these details are crucial to discuss later in the discussion section<br><br>See line 58 to 194
**Methods**	Sampling methods to my view is not correct, if you used stratified sampling what were the strata? generally the participants section should be revised extensively by explaining the more and correct details about the sampling process	Have added a clarification from line 203 to 212
	instruments<br>Total scales wrong term?	Have replaced it with a correct term, see line 243

	DERS-18 should be explained to the readers with more details from developers.	Have added an explanation From line 226 to 245
	More details about psychometric properties of DASS-21 from Malaysia are needed	Have added an explanation<br><br>From 246 to 264
	you explained very brief information about psychometric evaluation in statistical analysis subsection, you should subdivide this section into subsections with heading such as "construct validity" , internal consistency and item analysis" , "criterion validity" and explain the process for each of above procedures with more details	Have added the subsection<br><br>From 320 to 364
	Surprisingly a very important step i.e. test retest reliability has been missed	The study was conducted during the Covid-19 pandemic, which may have affected the emotional state of adolescents over time. A test-retest study was not conducted as it was deemed impractical. The data collection was also affected by the pandemic, as some students may have been absent from classes due to various reasons, making it difficult to arrange for a re-test session. Additionally, academic activities were disrupted due to the pandemic, making it harder to arrange multiple meetings.
	Ceiling and floor effect evaluation have been missed	Have added the evaluation<br><br>From line 326 to 340
	How about the process of face and content validity evaluation and their results	Have added explanations on the process of evaluating the face and content validity<br><br>See line 277 to 294<br><br>The results of these validity are shown in line 367 to 385

**Results**	For all above points i strongly recommend authors to see published papers on psychometric evaluation in good refereed journals for obtaining guides on how to write and complete the above mentioned shortfalls	Have reviewed the published papers on psychometric evaluation especially papers from PLOS One
	I is expected to present a table explaining the study participants characteristics including basic such as age, gender, education,...	Have added the description of participant characteristics<br><br>See line 387 to 396<br><br>We also added a table (Table 1) at line 399
	and the mean scores of instruments subscales you used in this study,	We have added a table (Table 2) at line 425
	Table 1 does not present informative data in the framework of psychometric analysis, particularly correlations	The table containing correlation has been excluded<br><br>See line 402 to 406
	The results about reliability analysis has major defects, the most important you did not follow the union approach for treating the awareness domain , you should follow the original version for scoring and then follow the reliability analysis, please revise and reconsider this very important point	We have followed the suggestion<br><br>See line 428 until 445
	Skewness Kurtosis are not informative data in this regard	Have excluded the information<br><br>See Table 3 at line 447
	Please present the results of test retest analysis	Due to the pandemic, it was deemed impractical to conduct a test-retest study and data collection was affected by student absences. Academic activities were also disrupted, making it challenging to arrange multiple meetings.
	Please (after reconsidering awareness domain) present CFA results in a suitable figure as is very common in this area of subject, the correct presentation as table is confusing	We have uploaded a figure (Figure 1) as an individual file that is separate from the manuscript<br><br>The figure will be inserted at line 463

	Please follow my point regarding the criterion validity in abstract, and see published papers for getting guides on how to present and interpret the results of criterion validity.	We have presented the results of criterion validity<br><br>For the concurrent validity, see line 483 to 492<br><br>For the predictive validity, see line 494 to 507
	Please present the results of Ceiling and flor effect evaluations.	Presented at line 408 to 423
